# *In-vitro* Evaluation of Cytotoxic and Apoptogenic Properties of *Sophora Pachycarpa*

**Published:** 2014

**Authors:** Seyed Hadi Mousavi, Mahsa Motaez, Amir Zamiri-Akhlaghi, Seyed Ahmad Emami, Zahra Tayarani-Najaran

**Affiliations:** a *Medical Toxicology Research Centre, Mashhad University of Medical Sciences, Mashhad, Iran. *; b *Pharmacological Research Centre of Medicinal Plants, School of Medicine, Mashhad University of Medical Sciences, Mashhad, Iran. *; c *Department of Pharmacognosy, School of Pharmacy, Mashhad University of Medical Sciences, Mashhad, Iran.*; d *Department of Pharmacodynamics and Toxicology, School of Pharmacy, Mashhad University of Medical Sciences, Mashhad, Iran. *

**Keywords:** Apoptosis, *Sophora pachycarpa*, Fabaceae, Cytotoxicity, Cancer

## Abstract

*Sophora pachycarpa *Schrenk ex C.A.Mey. belongs to the family Fabaceae. Some species of the genus *Sophora *have shown to possess anti-proliferative and apoptosis-inducing activities in cancer cells. However, there is no available information addressing this effect in *S. pachycarpa*. Here, we investigated the cytotoxic effects of methanol extract and different fractions obtained from *S. pachycarpa *root on different cancer cell lines including A549, HeLa, HL-60, MCF-7, and PC3 cell lines and leukocytes as non-malignant cells. Apoptotic cells were determined using PI staining of DNA fragmentation by flow cytometry (sub-G1 peak). *S. pachycarpa *inhibited the growth of malignant cells in a dose-dependent manner. CH_2_Cl_2_ and EtOAc fractions showed the lowest IC_50_ values ranging from 6 to 50 μg/mL in various cancer cell lines. HeLa cells as the most sensitive cells were chosen for further mechanistic studies. The sub-G1 peak in flow cytometry histogram of* S. pachycarpa *treated HeLa cells indicates apoptotic cell death in *S. pachycarpa*-induced toxicity. In conclusion, *S. pachycarpa *exerts cytotoxic effects in different cancer cell lines in which apoptosis plays an important role. Thus, *S. pachycarpa *could be considered as a potential chemotherapeutic agent in cancer treatment.

## Introduction

Plants have been served as the main sources of biologically active compounds in the prevention as well as treatment of many diseases including cancer. Molecular diversity of bio-active compounds in natural products provides diverse and wealthy source of phytochemical with recognized potential in drug discovery and development (1,2). Numerous cytostatic drugs with antitumor properties are plant derived components including alkaloids, phenolics, flavonoids, and terpenoids (3). 

The genus *Sophora *(Fabaceae) is divided into two subgenera, *Sophora* and *Styphnolobium*. This genus has 52 species in the world and three of these species are found in Iran include: 1-* S. mollis* (Royle) Baker with three subspecies *mollis*, *griffithii *(Stocks) Ali and *sylves* M. Abbasi; 2- *S. alopecuroides* L. with two subspecies *alopecuroides* and *tomentosa* (Boiss) Yakovlev; and 3- *S. pachycarpa *Schrenk ex C.A.Mey, and a hybrid *S. alopecroidese* × *S. pachycarpa* (4). 

The dried roots of *Sophora* are one of the most popular and multi-purpose herbs used in China [*S. tonkinensis* (Pierre) Craib ex Hartwiss] and Japan [*S. flavescens *Aiton] (5). 

The root of *S. flavescens *is a popular Chinese herbal medicine (Chinese name ‘‘Ku-Shen’’) used in a variety of disorders including gastric disturbance and eczema, and also as anti-pyretic and anti-helmentic. This genus is an abundant source of pernyl flavonoids (5-11), flavonoids (12) and alkaloids (13-17). 

Previous studies on the chemical composition of *S. pachycarpa* showed the presence of quinolizidine alkaloids (18,19), flavonoides and steroid glucosides (12). *Sophora* alkaloids have been found to be the chief active component of the plant including matrine, oxymatrine, sophocarpine, sophoramine, and sophoridine. Basic and clinical researches have confirmed that these alkaloids possess different pharmacological effects like anti- inflammatory (20,21), immunity-regulatory (22), antiviral (23), antibacterial (24), and anti-tumor properties (25,26). From the published data mentioned above genera and families, it is possible to predict the type of compounds that might be present in a particular extract. This tentative prediction on possible identity of the classes of compounds may help choose suitable extraction and partitioning methods and appropriate solvents for extracting specific classes of compounds (27,28). In this study, the uses of solvents for screening and the isolation of active components were examined. 

Despite several reports on the anti-cancer properties of some species of the genus, *S. pachycarpa* has not yet been studied for its cytotoxic potential. 

Cancer is considered as the leading cause of death in developed countries and the second leading cause of death in developing countries. In order to evaluate new compounds and extracts for potential *in-vitro *activity in the primary screening model, employing diverse panel of human tumor cell lines representing major tumor types is recommended. Evaluations may carry out on compounds over a wide range of concentrations for cytotoxic or growth-inhibitory effects against each cell line comprising the panel. Lung cancer (1.4 million deaths) is the most common cause of death in patients suffering from cancer worldwide (29). A549 is a cell line derived from human alveolar cell carcinoma (30) and has been used as model of both human primary alveolar epithelial cells (31) and human primary alveolar epithelial cells *in-vitro *(32). Over the course of a lifetime, 1 in 8 women will be diagnosed with breast cancer. MCF-7 is a human breast cancer cell line, extensively used in the study of breast cancer (33). Cervical cancer is the third most common type of cancer in women (34). HeLa cells are human epithelial cells studied widely in cervical cancer, the second most frequent malignant tumor in women (35). Prostate cancer is the most common cause of death from cancer in men over age 75 (36). PC3 is the classic human cell line of prostatic cancer (37). This cell line was used in studies evaluating the response of chemotherapeutic drugs on prostate cancer cells (38). The third most common childhood cancers is leukemia (34%) (39). The HL-60 (Human promyelocytic leukemia cells) cell line is a leukemic cell line which is used to study the apoptosis effect of the extracts on cells (40). 

Here we analyzed for the first time the cytotoxic effects of *S. pachycarpa *root extract on different cancer cell lines including A549, HeLa, HL-60, MCF-7, PC3 cells, and human umbilical cord Leukocyte as non-malignant cells. The role of apoptosis in *S. pachycarpa-*induced cytotoxicity on cancer cell lines was explored as well. 

## Experimental

*Reagents and chemicals*

The fluorescent probe propidium iodide (PI), and Triton X-100 were purchased from Sigma (St Louis, MO, USA), MTS from Promega (Madison, WI, USA), RNase A and Proteinase K from Fermentas (Vilnius, Lithuania); RPMI-1640 and FCS from Gibco (Grand Island, USA). 

*Plant materials*

Roots of *S. pachycarpa *were collected in June 2010 at the Ferdowsi University Campus, Mashhad, Razavi Khorasan province, northeast of Iran. 

The plant was identified by Mr. M. R. Joharchi from Ferdowsi University of Mashhad Herbarium (FUMH). A voucher specimen (No. 06-019-016) is deposited in the herbarium of the School of Pharmacy, Mashhad University of Medical Sciences, Mashhad, Iran. 

**Figure 1:**
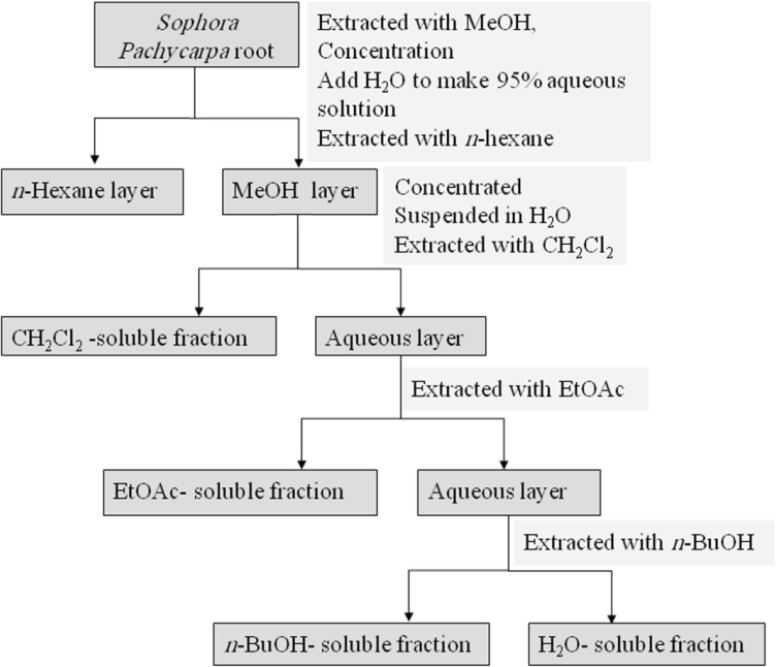
Partitioning scheme using immiscible solvents

The dried root (380 g) was percolated with methanol (MeOH) at room temperature. The whole extract was filtered and the solvent was evaporated under reduced pressure at 40-45°C, to afford crude methanol extract. Methanol extract was then resolved in MeOH: H_2_O 95:5 and partitioned successively between *n*-hexan, dichloromethane (CH_2_Cl_2_), ethylacetate (EtOAc), *n*-butanol (*n*-BuOH), and finally H_2_O. CH_2_Cl_2_, EtOAc, and *n*-BuOH fractions were evaporated under vacuum and H_2_O fraction was freeze dried to 0.4, 5.2, 3.5, and 5.9 g north of respectively. Fractions were stored at 4°C until analysis. A partitioning scheme of *S. pachycarpa *methanol extract is presented in Figure 1 (27). 

*Sample preparation*

To prepare the stock solutions (100 mg/mL), each extract was dissolved in DMSO. The concentrations of 3.5-250 *µ*g/mL were then obtained by diluting these solutions with Roswell Park Memorial Institute-1640 (RPMI-1640) so that the final concentrations of DMSO did not exceed 0.25%. All dilutions were prepared fresh before addition to the cells. 

*Cell culture and Treatment*

A549, HeLa, HL-60, MCF-7, and PC3 cells were obtained from Pasteur Institute (Tehran, Iran) and maintained at 37ºC in a humidified atmosphere (90%) containing 5% CO_2_. Cell lines were cultured in RPMI 1640 supplemented with 10% (v/v) fetal bovine serum, 100 U/mL penicillin and 100 mg/mL streptomycin. Cells were seeded overnight and then incubated with various concentrations of different extracts for 48 h. 

For MTS assay, cells were seeded at 10^4^ cell per well onto 96-well culture plates. For assay of apoptosis, cells were seeded at 10^5^ cell per well onto a 24-well plate. 

For each concentration and time course study, there was a control sample which remained untreated and received an equal volume of the solvent. Paclitaxel (41) was used as positive control (350 nM). 

*Cell viability*

The MTS assay (42) is based on the reduction of MTS by mitochondrial dehydrogenases in metabolically active cells, to form the colored, water-soluble formazan. Cells were seeded in each well of a 96-microwell plate and treated with various concentrations of methanol extract and different fractions obtained from *S. pachycarpa *root. After incubation for 48 h, CellTiter 96® Aqueous One Solution Reagent (Promega, Madison, WI, USA), which is composed of the novel tetrazolium compound MTS and an electron coupling reagent phenazine methosulfate (PES, a redox intermediary), was added to each well according to the manufacturer’s instructions. After 1 h, the cell viability was determined by measuring the absorbance at 490 nm using an ELISA microplate reader (Awareness, Palm City, FL, USA). 

Cytotoxicity was expressed as IC_50_, which was calculated using Graph Pad prism 5 software and presented as mean ± SEM of three independent experiments with three replicates for each concentration. 

Paclitaxel was used as a positive control. 

*Leukocyte culture*

Human umbilical cord blood samples (50 ml) were collected from a fresh umbilical cord attached to the placenta by gravity flow in sterile 50 ml syringe containing citrate buffer as an anticoagulant. The sample was diluted with an equal volume of Phosphate Buffered Saline (PBS), layered over Ficoll-Hypaque solution according to density gradient (1.077 g/mL), and centrifuged at 800 g for 20 min at room temperature. The mononuclear cell layer was removed, washed twice in PBS and resuspended in RPMI 1640 medium supplemented with 10% (v/v) fetal bovine serum, 100 U/mL penicillin and 100 mg/mL streptomycin. Leukocytes (5×10^4^ cells per well) were treated with different concentrations of each fraction of *S. pachycarpa* in 96-well plates, for 48 h. This study protocol was approved by the ethical committee of Mashhad University of Medical Sciences. 

*Apoptosis*

Apoptotic cells were detected using PI staining of treated cells followed by flow cytometry to detect a sub-G1 peak (43,44). 

It has been reported that DNA fragmentation creates small fragments of DNA that can be eluted following incubation in a hypotonic phosphate citrate buffer. When stained with a quantitative DNA-binding dye such as PI, cells that have lost DNA will take up less stain and will appear to the left of the G1 peak. Briefly, HeLa cells were cultured overnight in a 24-well plate and treated with various concentrations of CH_2_Cl_2_ extract for 48 h. Floating cells were harvested and incubated at 4°C overnight in the dark with 750 µL of a hypotonic buffer (50 µg/mL PI in 0.1% sodium citrate + 0.1% Triton X-100) before flow cytometric analysis using a Partec flow cytometer (GmbH, Münster, Germany) was conducted. Ten thousand events were acquired. 

*Statistics*

One-way analysis of variance (ANOVA) and Bonferroni’s post hoc were used for data analysis. All results were expressed as mean ± SEM and p-values <0.05 were considered statistically significant. 

## Results

*Inhibition of cell viability*

**Figure 2:**
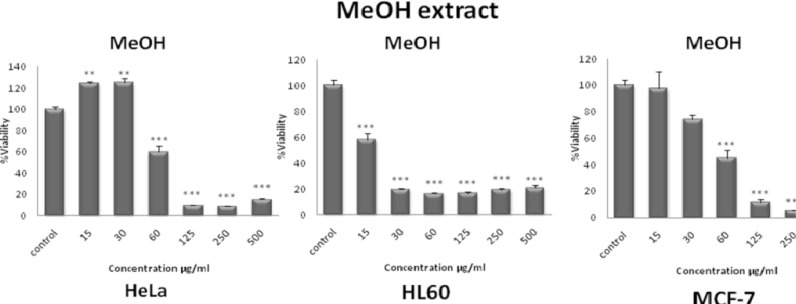
Dose-dependent growth inhibition of malignant cell lines by total methanol extract (15-500 μg/mL) after 48 h. Viability was quantitated by MTS assay. The toxicity started at a concentration as little as 15 μg/mL. Results are mean ± SEM (n =9). *p < 0.05, **p < 0.01 and ***p < 0.001 compared to control

The total methanol extract of *S. pachycarpa* decreased cell viability in a concentration-dependent manner. The toxicity was first observed at a concentration lower than 15 µg/mL (Figure 2). IC_50 _values for total methanol extract of *S. pachycarpa *in each cell line are presented in Table 1. 

**Table 1:** Doses of total methanol extract of *S. pachycarpa *inducing 50% cell growth inhibition (IC_50_) against malignant cell lines. Cells incubated with different concentration of extracts for 48 h

	**Cell line**		
	HeLa	HL-60	MCF-7
IC_50_	84.07	15.07	52.33

**Figure 3:**
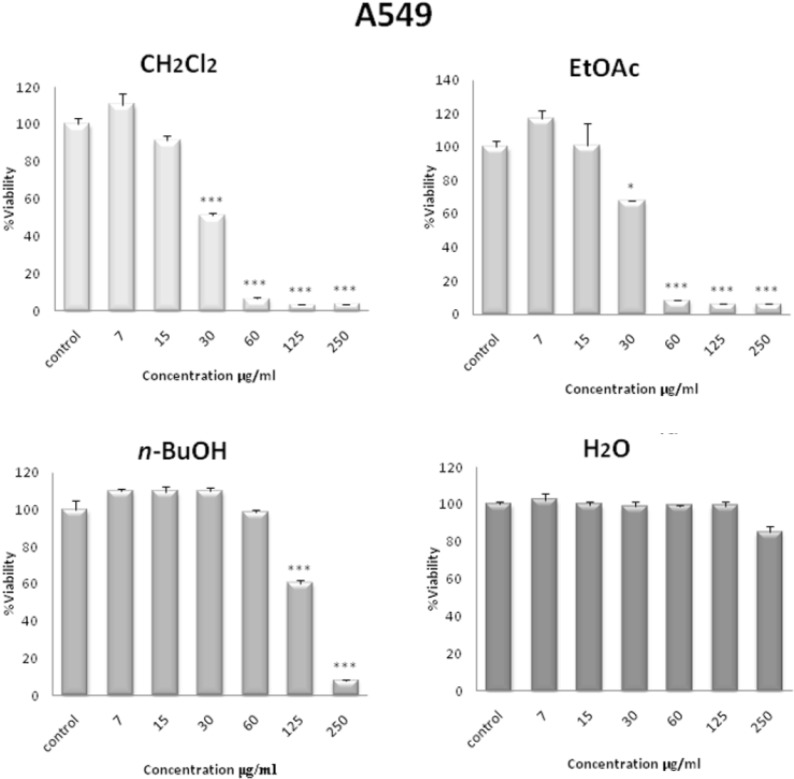
Dose-dependent growth inhibition of malignant cell lines by CH_2_Cl_2_ fraction (3.5-250 μg/mL) after 48 h. Viability was quantitated by MTS assay. Results are mean ± SEM (n =9). *p < 0.05, **p < 0.01 and ***p < 0.001 compared to control

In order to compare the cytotoxicity of solvent fractions of *S. pachycarpa*, another MTS assay was carried out for different concentrations (0-250 µg/mL). Among all the fractions tested, the CH_2_Cl_2_ and EtOAc fractions were found to be more effective than the other fractions of the plant extract. CH_2_Cl_2 _fraction had the maximum inhibitory effect on the proliferation of HL-60 cells (Figure 3). In contrast, the H_2_O fraction did not show any anti-proliferative effect. CH_2_Cl_2 _and EtOAc fractions effectively inhibited the growth of A549, HeLa, HL-60, MCF-7, as well as the PC3 cells in a concentration-dependent manner after 48 h. The IC_50_ values of solvent fractions of *S. pachycarpa *against malignant and non malignant cell lines are presented in Table 2. 

**Table 2:** Doses of solvent fractions of *S. pachycarpa *inducing 50% cell growth inhibition (IC_50_) against malignant and non malignant cell lines. Cells incubated with different concentration of extracts for 48 h

**Cell line**	**Fraction**			
	CH_2_Cl_2_	EtOAc	*n*-butanol	H_2_O
A549	31.15	40.53	189.3	>250
HeLa	14.54	26.62	86.20	>250
HL-60	10.52	6.651	35.29	>250
PC-3	43.22	233.2	72.17	>250
MCF-7	27.09	42.08	206.7	>250
Leukocyte (normal cells)	87.29	25.16	55.78	>250

In comparison, the cytotoxic effect of CH_2_Cl_2_ fraction on normal leukocyte proliferation isolated from peripheral blood was minimal (Figure 3). 

We used 350 nM of Paclitaxel as a positive control. Paclitaxel at this concentration decreased the viability of HeLa cells to 25.25% ± 5.58 (data not shown). 

Together, these data point to the selective activity of CH_2_Cl_2_ and EtOAc fractions against tumor cells. 

*Apoptosis induction by CH*_2_ *Cl*_2_ *fraction of S. pachycarpa*

**Figure 4:**
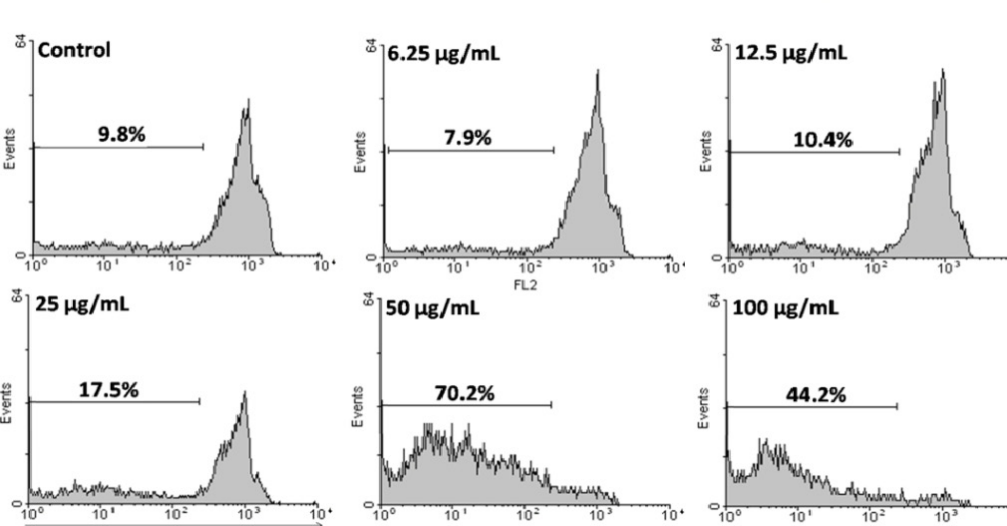
Flow cytometry histograms of apoptosis assays by PI method in HeLa cells. Cells were treated with different concentration of CH_2_Cl_2_ fraction for 48 h. Sub-G1 peak as an indicative of apoptotic cells, was induced in CH_2_Cl_2_ fraction treated but not in control cells. CH_2_Cl_2_ fraction-treated cells exhibited a sub-G1 peak in HeLa cells in a concentration dependent manner that indicates the involvement of an apoptotic process in CH_2_Cl_2_ fraction-induced cell death

HeLa cells as the most sensitive cells were chosen for further mechanistic studies. To confirm that the CH_2_Cl_2_ fraction of *S. pachycarpa *induces apoptosis, we evaluated the percentage of apoptotic cells by PI staining and flow cytometry, to observe sub-G1 peak caused by DNA fragmentation. CH_2_Cl_2_ fraction treated cells showed sub-G1 peak in HeLa cells. This indicates that CH_2_Cl_2_ fraction induces cell death through apoptosis (Figure 4).

## Discussion

Plants as the main source of the potent phytochemicals have been used to prevent and treat diseases including cancer for years. In this study, the cytotoxic and proapoptotic effects of methanol extract and its different fractions obtained from *S. pachycarpa *root extract were investigated on A549, HeLa, HL-60, MCF-7, and PC3 cells. Leukocytes were used as non-malignant cells while Paclitaxel (350 nM) was used as a positive control. 

To the authors’ knowledge, this is the first report on *S. pachycarpa* induced cytotoxicity in cancer cell lines. Our data confirmed that *S. pachycarpa *extract has cytotoxic effect on various tumor cell lines tested. 

Using MTS assay, the anti-proliferative effects of methanol extract and fractions of* S. pachycarpa *root extract on different cancer cell lines were compared. The effects of extracts and isolated fractions were not all anti-proliferative. Data showed that the CH_2_Cl_2_ fraction of *S. pachycarpa *has a significant cell growth inhibitory effect. 

Similarly, a study to find bioactive compounds from plants, the CH_2_Cl_2_ fraction of the MeOH extract of the root of *S. flavescens, *showed significant cytotoxicity against human myeloid leukemia HL-60 cells (14,45). 

Methanol extracts of* S. alopecuroides* and *S. pachycarpa* showed the LC_50_ values of 73.11 ± 1.20 and 56.73 ± 0.55 (μg/mL) in Brine shrimp lethality assay (46).

Herbal extract contains many components; different compounds may have opposing pharmacological activities. To ensure safe use of herbal medicine, these compounds with opposing effects should be removed from the extract. 

In the present study, the purification by solvent extraction of *S. pachycarpa *was used and the potential antitumor activity of low-polar solvent fraction (CH_2_Cl_2_, EtOAc) was compared to polar solvent fractions (*n*-BuOH and H_2_O soluble). 

The presence of compounds with synergistic or antagonistic interactions in crude extract may cause varying level of activity in total extract of the plant compared to its fractions. Differences in the activity of polar and non-polar fractions might be due to the presence of diverse constituents. The maximum activity of low-polar solvent fraction (CH_2_Cl_2_, EtOAc) can serve to trace the highly active phytochemicals. Sharing various phytochemicals in different fractions might be responsible for the change in the activity either due to synergistic or antagonistic effects. 

The chemo-physical properties of different compound in the extract can be exploited to initially divide them into various chemical groups using solvents of increasing polarity. 

The CH_2_Cl_2_ fraction as the most cytotoxic fraction of *S. pachycarpa* extract was further analysed to establish its apoptotic activity in the HeLa and HL-60 cell lines. 

Treatment of tumors is directed not only on inhibition of cell proliferation, but also on induction of apoptosis of tumor cells. 

It is well known that the earliest recognized morphological change in apoptosis is compaction and segregation of the nuclear chromatin, resulting in chromatin margination and cytoplasmic condensation. Condensation is accompanied by sinking followed by disintegration of the nucleus into discrete fragments, a characteristic feature of apoptosis involving endonucleases (47). 

This characteristic oligonucleosomal DNA degradation (also known as low molecular weight (LMW) cleavage) and chromatin condensation are among the best known events of apoptotic execution and were initially assumed to be an essential part of the apoptotic pathway (48). 

The CH_2_Cl_2_ fraction of *S. pachycarpa *possesses the ability to reduce the viability of HeLa cells via apoptosis induction confirmed by sub-G1 peak in the flow cytometry histogram of treated cells compared to control cells. 

Our results showed that CH_2_Cl_2_ fraction of *S. pachycarpa* inhibits cell growth via apoptosis. On the basis of these findings, we predict that the CH_2_Cl_2_ and EtOAc fractions of *S. pachycarpa* would be good candidates to search for novel bioactive components and their application as anti-cancer potential. 

Collectively, further studies are required to evaluate and understand the underlying molecular mechanisms, pathways involved and to identify active phyto-chemicals before* S. pachycarpa *being considered as a potential therapeutic agent against cancer. 
